# Retraction Note: Structural modeling of EFL/ESL teachers’ physical activity, mental health, psychological well-being, and self-efficacy

**DOI:** 10.1186/s40359-025-02814-w

**Published:** 2025-05-08

**Authors:** Min Guo, Shaohua Jiang

**Affiliations:** 1 School of Foreign Languages, Xinyang Normal University, Xinyang, 464000 China; 2 College of Foreign Languages, Fujian Normal University, Fuzhou, 3501007 China; 3 School of Humanities, Fujian University of Technology, Fuzhou, 350118 China; 4 Krirk University, Bangkok, 10220 Thailand

**Retraction Note: BMC Psychology (2023) 11:343**

10.1186/s40359-023-01383-0

The editor and the publisher have retracted this article. The article was submitted to be part of a guest-edited issue. An investigation by the publisher found a number of articles, including this one, with a number of concerns, including but not limited to compromised editorial handling and peer review process, inappropriate or irrelevant references or not being in scope of the journal or guest-edited issue. Based on the investigation’s findings the editor therefore no longer has confidence in the results and conclusions of this article.

The authors have not responded to correspondence regarding this retraction.

